# Multi-Bandgap Monolithic Metal Nanowire Percolation Network Sensor Integration by Reversible Selective Laser-Induced Redox

**DOI:** 10.1007/s40820-021-00786-1

**Published:** 2022-01-25

**Authors:** Junhyuk Bang, Yeongju Jung, Hyungjun Kim, Dongkwan Kim, Maenghyo Cho, Seung Hwan Ko

**Affiliations:** 1 Applied Nano and Thermal Science Lab, Department of Mechanical Engineering, Seoul National University, 1 Gwanak-ro, Gwanak-gu, Seoul, 151-742 Republic of Korea; 2 Department of Mechanical Engineering, Seoul National University, 1 Gwanak-ro, Gwanak-gu, Seoul, 08826 Republic of Korea; 3 Institute of Advanced Machines and Design, Institute of Engineering Research, Seoul National University, Seoul, 08826 Republic of Korea

**Keywords:** Monolithic integration, Seamless interface, Multi-bandgap nanowire, Laser-induced redox, Multispectral photodetector

## Abstract

Active electronics are usually composed of semiconductor and metal electrodes which are connected by multiple vacuum deposition steps and photolithography patterning. However, the presence of interface of dissimilar material between semiconductor and metal electrode makes various problems in electrical contacts and mechanical failure. The ideal electronics should not have defective interfaces of dissimilar materials. In this study, we developed a novel method to fabricate active electronic components in a monolithic seamless fashion where both metal and semiconductor can be prepared from the same monolith material without creating a semiconductor–metal interface by reversible selective laser-induced redox (rSLIR) method. Furthermore, rSLIR can control the oxidation state of transition metal (Cu) to yield semiconductors with two different bandgap states (Cu_2_O and CuO with bandgaps of 2.1 and 1.2 eV, respectively), which may allow multifunctional sensors with multiple bandgaps from the same materials. This novel method enables the seamless integration of single-phase Cu, Cu_2_O, and CuO, simultaneously while allowing reversible, selective conversion between oxidation states by simply shining laser light. Moreover, we fabricated a flexible monolithic metal–semiconductor–metal multispectral photodetector that can detect multiple wavelengths. The unique monolithic characteristics of rSLIR process can provide next-generation electronics fabrication method overcoming the limitation of conventional photolithography methods.

**Supplementary Information:**

The online version contains supplementary material available at 10.1007/s40820-021-00786-1.

## Highlights

- A three single-phase Cu, Cu_2_O, and CuO monolithic nanowire network was successfully fabricated by reversible selective laser-induced redox (rSLIR)
- Monolithic metal–semiconductor–metal multispectral photodetectors with Cu nanowire (CuNW) as an electrode and Cu_2_ONW/CuONW having different bandgaps were suggested.

**Supplementary Information:**

The online version contains supplementary material available at 10.1007/s40820-021-00786-1.

## Graphical abstract

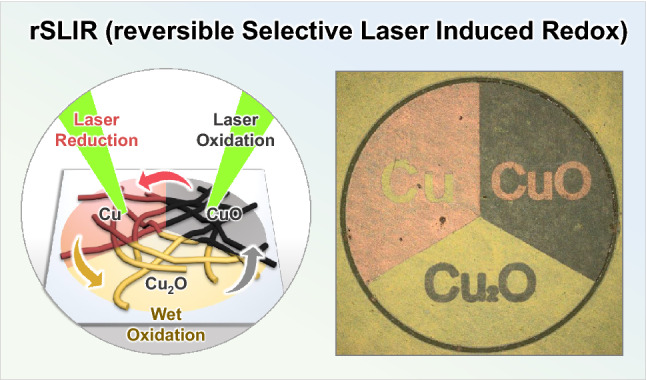

## Introduction

Changing and controlling electrical properties as desired are crucial for the manufacturing of electronic devices. Transition metals with incompletely filled *d*-orbitals show variable oxidation states: different states form depending on oxidation conditions and different characteristics present [1]. Transition metal oxides have achieved complex functionality and high performance in diverse fields such as sensors [2, 3], memory devices [4, 5], and energy devices [6, 7]. Nevertheless, sophisticated arrangements and integration of multiple oxidation states are some of the challenges left unconquered.

Copper, an essential electronic material in nanotechnology, is found in a metallic state and two distinct stoichiometric oxidation states in nature: Cu (zero valent), Cu_2_O (monovalent), and CuO (bivalent). Cu has been intensively investigated for their high electrical conductivity and abundant reserves. As p-type semiconductors, Cu_2_O and CuO have bandgaps of 2.1 and 1.2 eV [8, 9]. Cu_2_O and CuO are also utilized in a broad range of applications, such as sensors [10, 11], energy devices [12, 13], chemical catalysts [14, 15], photocatalysts [16, 17], and biomedical devices [18, 19]. One-dimensional copper nanowire (CuNW) with a high aspect ratio has attracted attention due to its inherent electric and mechanical characteristics [20, 21]. However, because of its high surface-to-volume ratio, nanowires are more readily degraded than bulk metal, more sophisticated oxidation conditions require investigation to manipulate single-phase Cu_2_O or CuO.

The laser process allows on-demand control of the oxidation state of transition metals, in contrast with conventional thermal oxidation, which is unavailable to elaborate patterns of multiple oxidation states entirely due to heat spreads. The focused laser illumination generates localized heat and exceeds the critical reaction temperature very quickly. Therefore, photothermochemical reaction enables another oxidation state that exhibits different electrical properties with seamless interfacing [22–29]. However, previous technologies are still limited to 0 D nanoparticle or incomplete three-phase control. They also have not been used for independent patterning of Cu, Cu_2_O, and CuO to nanowire networks.

This work presents reversible, selective laser-induced redox (rSLIR) of a three-single phase Cu, Cu_2_O, and CuO monolithic nanowire network. We achieved three materials patterning, including one metal and two metal oxides. rSLIR combines the three processes that cross over the oxidation state of Cu-based nanowires; it is simple yet allows complex materials patterns. The prepared CuNW network was oxidized under low temperature and high humidity, transforming it into Cu_2_ONW networks. Afterward, laser irradiation of the Cu_2_ONW network selectively induced oxidation or reduction, obtaining a monolithic, seamless CuNW, Cu_2_ONW, and CuONW network. rSLIR constituted a 3-phase cycle from synthesized CuNW to wet-oxidized Cu_2_ONW, laser-oxidized CuONW, and laser-reduced CuNW. The 3-phase cycle did not restrict the starting state of the nanowire network during rSLIR. We demonstrated that rSLIR could be conducted on nanowire networks in which Cu and Cu_*x*_O are irregularly mixed, assuring their reversibility. Moreover, to verify that our method is suitable for multiple disparate electronic device fabrication, we suggested using an MSM (metal–semiconductor–metal) visible light photodetector with CuNW as an electrode and Cu_2_ONW and CuONW, having different bandgaps, in the visible light detecting channels.

## Experimental Section

### Synthesis of CuNW

Hydrothermal synthesis was implemented for CuNW. Copper (II) chloride dihydrate (Sigma-Aldrich, No. 467847) was the precursor, HDA (Sigma-Aldrich, No. 445312) was the ligand, and glucose (Sigma-Aldrich, No. 5767) was used as a reducing agent. 0.84 g of CuCl_2_ and 5.2 g of HDA were dissolved in 400 mL of deionized water with vigorous stirring. Next, 2 g of glucose was added to the prepared solution and reacted at 100 °C for 7 h and 20 min. During the growth of nanowire, the color of the CuNW solution change to reddish-brown. Last, centrifugation for 15 min at 1500 rpm was performed with IPA several times to purify CuNW solution. The synthesized CuNW is 80–100 thick and 80–110 µm long and is highly conductive with a sheet resistance of 50 Ω sq^−1^ at 89% transparency in Fig. S1.

### Wet Oxidation to Produce Cu_2_ONW

The CuNW network became a Cu_2_ONW network through wet-oxidation with mild temperature conditions, as shown in Fig. S2. First, a uniform CuNW network was obtained through vacuum filtration. After that, the red CuNW network was initially oxidized for 12 h at 100 °C with high humidity. The sample turned dark red, and at this time, only a weak copper oxide peak was detected by XRD due to its insufficient oxide grain sizes. Next, the dark red Cu_*X*_ONW network was oxidized in one day at room temperature, and high relative humidity (over 80%), turning from dark red to bright yellow, indicated a single oxidation state of Cu_2_ONW.

### DFT Calculations

Spin-polarized density functional theory (DFT) calculations were performed using density functional theory as implemented in the Vienna ab initio simulation package [30, 31], using the projector augmented wave with the Perdew–Burke–Ernzerhof generalized gradient approximation (GGA) exchange–correlation function [32–34]. An energy cutoff of 520 eV was used for all calculations. The valence configurations of the Cu and O elements are 3*p*^6^3*d*^10^4*s*^1^ and 2*s*^2^2*p*^4^, respectively. The Cu_2_O(111) (2 × 1) slab was constructed based on the fully relaxed Cu_2_O bulk unit cell, and the optimized O_2_ molecule was positioned on the Cu_2_O(111) substrate to make the interfacial nanostructure. Monkhorst–Pack *k*-point sets were used for all the calculations on the Cu_2_O(111) (2 × 1) slab model, 1 × 4 × 1. The climbing image nudged elastic band (CI-NEB) method was used with the limited-memory Broyden–Fletcher–Goldfarb–Shanno (L-BFGS) optimizer until the residual forces were below 0.05 eV Å^−1^ to calculate the reaction barriers for Cu_2_O oxidation [17, 35, 36]. The adsorption energy was computed to determine the energetically favorable O_2_ molecule adsorption site on Cu_2_O(111) surface following [37]:

1 $$ E_{{{\text{ads}}}} = E_{{{\text{O}}_{2} - {\text{Cu}}_{2} {\text{O}}\left( {111} \right)_{{{\text{surf}}}} }} - E_{{O_{2} }} - E_{{{\text{Cu}}_{2} {\text{O}}\left( {111} \right)_{{{\text{surf}}}} }} $$

where $E_{{{\text{ads}}}}$ is the adsorption energy and $E_{{{\text{O}}_{2} - {\text{Cu}}_{2} {\text{O}}\left( {111} \right)_{{{\text{surf}}}} }}$, $E_{{{\text{O}}_{2} }}$, and $E_{{{\text{Cu}}_{2} {\text{O}}\left( {111} \right)_{{{\text{surf}}}} }}$ are the total energy of the O_2_ molecule absorbed on the Cu_2_O(111) surface, the O_2_ molecule, and the bare Cu_2_O(111) surface obtained from the first-principles calculations, respectively.

### Finite Element Analysis

Finite element analysis was conducted to simulate homogenous photothermal effect during the laser oxidation process of Cu_2_ONW (Comsol Multiphysics). In the simulation, highly percolated Cu_2_ONW network locates on a SiO_2_ substrate, and the laser beam illuminates on the Cu_2_ONW perpendicularly from the top. According to the Cu_2_ONW used in the experiments, a Cu_2_ONW was modeled as a cylinder with a diameter of 100 nm and a length of 100 μm. Each Cu_2_ONW was placed 2 μm apart, and the same arrangement was stacked by rotating 90 degrees to reflect the overlapping of Cu_2_ONW. We also considered a Gaussian laser beam with a width of 20 μm and a power of 40 mW.

### Characterization

Scanning electron microscope (SEM, MERLIN Compact), X-ray diffraction (XRD, D8 Advance, 2020), and Raman spectroscopy (Renishaw InVia Raman microscope) were used to characterize the morphology and composition according to the phase transition of CuNW, Cu_2_ONW, and CuONW.

### Fabrication of Flexible Photodetectors

In this study, a 532 nm visible light laser (Sprout‐G‐5W, Lighthouse Photonics, USA) and Galvano-mirror (hurrySCAN II, Scanlab, Germany) were used for laser patterning. Figure S3b describes the fabrication steps. (1) For the Cu–Cu_2_O–Cu photodetector, spaced laser reduction hatch scanning produced CuNW electrodes with a Cu_2_ONW detecting channel 30 µm wide. (2) For the Cu–CuO–Cu structure, a CuONW detecting channel was produced through laser oxidation, and then CuNW electrodes were accomplished through laser reduction. (3) Cu–Cu_2_O&CuO–Cu, firstly, CuONW was fabricated by laser oxidation. After, laser ablation 40 separate CuONW and Cu_2_ONW detecting channels with 40 µm width were formed. Next, CuNW electrodes were formed on both sides in the same way as above. Laser ablation of the photodetector surroundings prevented electrical interference associated with external Cu_2_ONW. Next, to fabricate a flexible photodetector, polyurethane acrylate (PUA, SEA-1, CCTech) was spin-coated on the photodetector at 500 rpm for 30 s. Last, the device was placed under a UV lamp for 120 s to cure the PUA, and the PUA-nanowire composite was obtained.

### Photodetector Measurement Setup

The single-wavelength laser beams (450, 532, and 650 nm) were expanded to fully cover the photodetector channel. The expanded beam was adjusted through the nd filter for achieving intensities of 5, 10, and 15 mW cm^−2^, and the photocurrent was measured.

## Results and Discussion

### Reversible Selective Laser-Induced Redox

**Fig. 1:**
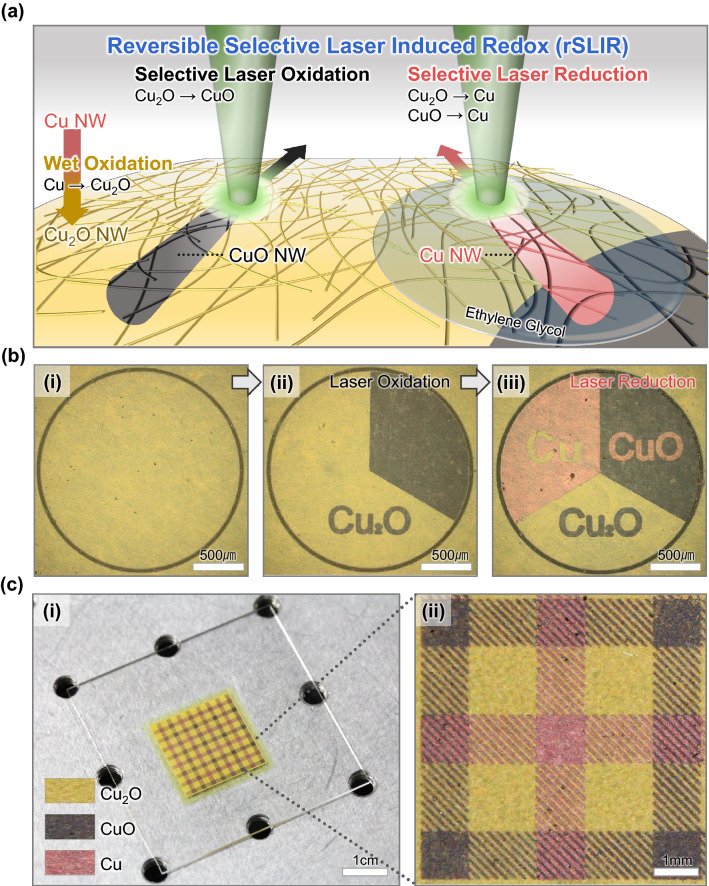
**a** Schematic of reversible selective laser-induced redox (rSLIR) for the monolithic CuNW, Cu_2_ONW, and CuONW network. **b** Optical microscopy image depicting the flow of rSLIR for **i** Cu_2_ONW **ii** Cu_2_O, and CuO patterns obtained after laser oxidation with 25 mW of power and a 1 mm s^−1^ scanning speed. **iii** Cu, Cu_2_O, CuO pattern obtained after laser reduction (scale bar: 500 µm). **c** Optical image of the sophisticated three-coloration pattern, **i** tartan check pattern (scale bar: 1 cm), **ii** magnified image of the tartan check pattern. The black CuONW and the red CuNW patterns were fabricated under ten cycles of the hatch scan at 125 mW power and 10 mm s^−1^ scan speed, and one cycle of the hatch scan at 125 mW power and 10 mm s^−1^ scan speed, respectively (scale bar: 1 mm)

The rSLIR schematic of the CuNW, Cu_2_ONW, and CuONW network is shown in Fig. 1a The continuous-wave laser (532 nm wavelength) irradiated the Cu_2_ONW networks (yellow color) fabricated through CuNW network wet oxidation. Laser-induced photothermal energy selectively generated oxidation or reduction according to the surroundings. Under ambient conditions, the photothermal energy led to selective oxidation, and CuONW (black color) developed. The laser enforced bonds between Cu_2_ONW and its adjacent oxygens and created another oxidation state, CuONW. When reducing agents surrounded the Cu_2_ONW network, the laser scan selectively reduced CuNW (red color). Ethylene glycol (EG), used for effective laser reduction, was dehydrated by photothermal energy, turning it into acetaldehyde, reducing the metal oxides [23, 38, 39].

rSLIR enables on-demand patterning of CuNW, Cu_2_ONW, and CuONW with seamless interfacing. The microscopic optical images in Fig. 1b describe the step-by-step fabrication of the delicate three single-phase pattern. Before the consecutive laser oxidation and reduction, we prepared a non-patterned 2 mm diameter Cu_2_ONW network disk through laser ablation (Fig. 1bi). Ten cycles of laser hatch scanning produced the black CuONW pattern (right circular arc and the letter of Cu_2_O) (Fig. 1bii). Applying EG on the Cu_2_ONW and CuONW pattern afterwards achieved a reddish CuNW pattern (left circular arc and the letter of CuO) with laser hatch scanning (Fig. 1biii). Last, the remnant EG was removed with ethanol. rSLIR usage can also be expanded to a large-scale pattern. A red-yellow-black (Cu, Cu_2_O, and CuO, respectively) tartan check pattern was manufactured through laser oxidation and reduction as 2 cm × 2 cm square Cu_2_ONW networks (Fig. 1ci, ii). Furthermore, rSLIR can be extended to the elastomeric stretchable substrate (Fig. S4).

During conventional thermal oxidation to form individual Cu_2_O and CuO, the high temperature and partial pressure of oxygen should be controlled [40]. Additionally, CuO is usually created after forming the Cu_2_O layer [41]. Likewise, during laser oxidation of Cu, incidental Cu_2_O is simultaneously formed at the edge of CuO and affected by the heat spreading. However, the wet oxidation for Cu_2_ONW before the laser process contributed to forming the single-phase pattern. The novel CuNW, Cu_2_ONW, and CuONW patterning process gave clear advantages over previous processes, including (1) a monolithic, seamless interface between individual oxidation state, (2) mask-less in-situ process, and (3) low-temperature and ambient conditions process.

### rSLIR for Monolithic CuNW, Cu_2_ONW, and CuONW Network

**Fig. 2:**
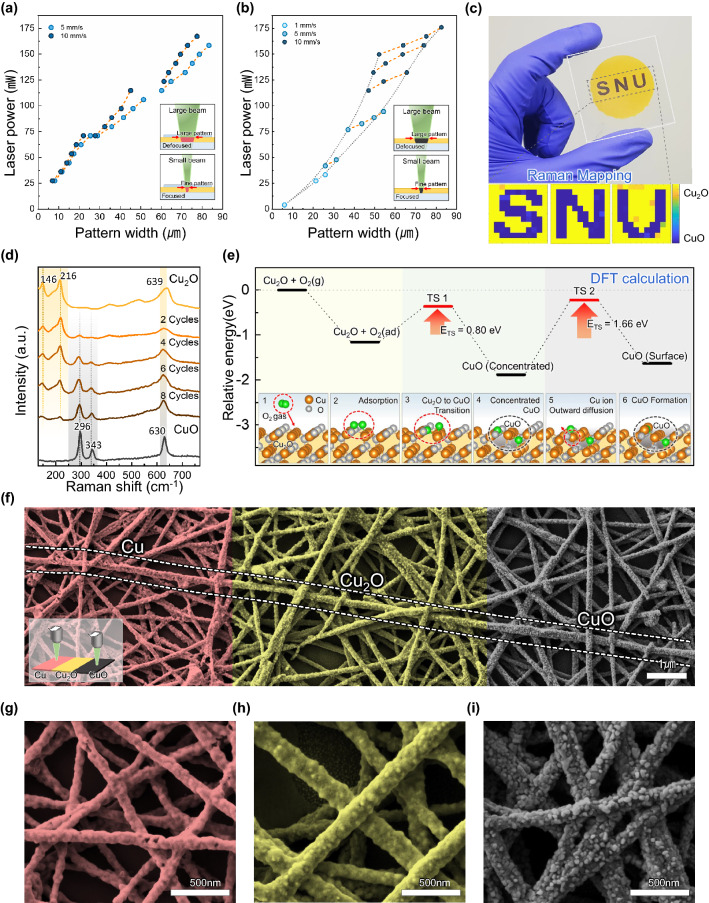
**a** An increase in laser-reduced CuNW pattern width according to laser power. **b** An increase in laser-oxidized CuONW pattern width according to laser power. **c** Optical image of Cu_2_ONW, CuONW pattern, and quantitative Raman mapping image investigated by 121 points data at 1 mm intervals (scale bar: 500 cm). **d** Raman spectroscopy of laser oxidation at different scan cycles at 125 mW power and 10 mm s^−1^ scanning speed. **e** DFT model for theoretical investigation of Cu_2_O to CuO transition. The six inset schematics depict the oxygen and copper atoms move as oxidation proceeds. **f** SEM image of monolithic CuNW, Cu_2_ONW, and CuONW network, the left red area Cu, central area Cu_2_O, and right area CuO. The inset schematic describes the process for making a monolithic structure (scale bar: 1 µm). **g** SEM image of CuNW. **h** SEM image of Cu_2_ONW. **i** SEM image of CuONW (scale bar: 500 nm)

In general, laser scanning fills the desired area through hatch scanning so that the pattern width is the practical and essential parameter. The laser power determined the amount of generated heat and affected the phase change width. Figure 2a shows the CuNW linewidth resulting from laser reduction by varying the laser intensity. The minimum resolution was obtained at 26 mW, and 10 mm s^−1^, and the power density was 67.6 Kw cm^−2^ (Fig. S5a). As the laser power increased, the width thickened, and excessive power produced a damaged pattern (Fig. S6a). Thus, to enlarge the pattern width without damage, the laser beam must be defocused like in the inset schematic in Fig. 2a. Figure 2b shows the available pattern width of CuONW through laser oxidation based on a ten-cycle laser scan. Laser oxidation obtains the narrowest line under the condition of 4 mW power, 1 mm s^−1^ scanning speed, and the power density at this time is 10.4 kW cm^−2^. (Fig. S5b) The nanowires melt and become damaged at high intensity, and the defocused beam achieves a thicker width line, as found earlier (Fig. S6b). In this regard, since laser beam size dominantly affects the pattern width, if the beam size can be further narrowed, the pattern width is likely to decrease.

Quantitative analysis of laser oxidation was completed using XRD and Raman spectroscopy. XRD analysis verified that wet-oxidized Cu_2_ONW, laser-oxidized CuONW, and laser reduced CuNW were single-phase (Fig. S7). To evaluate the crystallinity of the materials, we calculate the full width at half maximum (FWHM) of the X-ray diffraction line of both CuONW and Cu_2_ONW in Table S1. Generally, the sharper the XRD peak, the better crystallinity materials have. Considering that the sharpness of the XRD peak and FWHM value have inverse proportion, the results indicate that CuONW has better crystallinity compared to Cu_2_ONW.

Raman spectroscopy is an excellent way to identify different oxide compositions and confirm the atomic arrangements of materials. Through surface-sensitive Raman analysis, it can be seen that oxidation occurs on the surface of the nanowire. Other XRD accurately detects when grains grow sufficiently, this Raman spectroscopy detects amorphous crystals proving that Cu_2_ONW and CuONW have single phases. According to the laser scan cycle for oxidation from Cu_2_O to CuO, the Raman peak change is analyzed in Fig. 2d. The spectrum clarifies the oxidation state change from Cu_2_ONW to CuONW. The initial Cu_2_ONW spectrum consists of 5 peaks (three intense peaks at 146, 216, and 639 cm^−1^, and two weak peaks at 415 and 529 cm^−1^). As the laser cycles were incremented, the Cu_2_O peaks (146 and 216 cm^−1^) gradually disappear, and simultaneously the CuO peaks (296 and 343 cm^−1^) increase. Eventually, after ten cycles of laser scanning, only the CuO peaks remain and the laser-oxidized CuONW does not possess Cu_2_ONW.

Raman mapping investigated the reliability of the Cu_2_ONW and CuONW pattern, shown in Fig. 2c. The ratio of the most intense peak of Cu_2_O (216 cm^−1^) and CuO (296 cm^−1^) validates that the CuO pattern was uniformly generated even on a large scale by laser oxidation. Also, in Fig. S8, the Raman spectrum at different points shows the high repeatability of rSLIR.

The laser scanning process was simulated using finite element analysis in Fig. 2e to thoroughly understand the Cu_2_ONW and Cu_2_ONW laser-induced oxidation. Figure S9 shows that the area exposed to the laser was heated, while the remaining area was at room temperature, indicating that the laser selectively raises the energy of the exposed area. Considering the energy provided by laser scanning, the Cu_2_O oxidation procedure was inspected by the first-principles calculations in detail. The first-principles calculations were conducted on the energetically most stable Cu_2_O (111) surface model (Fig. S9 and Table S2) with a free O_2_ molecule on it. All possible O_2_ adsorption sites on the Cu_2_O (111) surface (Fig. S10 and Table S3) were examined, and the energetically stable site was considered in all following calculations to explore the potential adsorption sites. Figure 2e inset 2 shows an O_2_ molecule adsorbs on a bridge site of copper atoms. The adsorbed O_2_ molecule then interacts with neighboring copper atoms to dissociate into two oxygen atoms and oxidize the periphery, as shown in the Fig. 2e inset 2–4. For further oxidation inside Cu_2_ONW, an oxygen atom moves inward, or a copper atom diffuses outward. Notably, it requires relatively lower energy for a copper atom inside the bulk to diffuse outward than the energy for the oxygen atom to go inside, so it is preferential for a copper atom to diffuse to the Cu_2_ONW surface for further oxidation [42]. Figure 2e inset 4–6 shows a copper atom inside the bulk Cu_2_ONW diffuses out to the surface and balances the copper concentration. The Cu_2_ONW oxidation process could be classified into two steps: (1) adsorption and dissociation of an oxygen molecule and (2) the diffusion of a copper atom toward the surface. For each step, the calculated activation energy barrier was 0.80 eV (TS1) and 1.66 eV (TS2), so that during the second step, the copper atom diffusion outward was the rate-determining step for Cu_2_ONW oxidation. The reaction for the rate-determining step requires relatively high activation energy and occurs inside the bulk. It is essential to deliver an appropriate amount of energy and expose it in a certain amount of time for proper oxidation. Therefore, laser oxidation was only possible with a continuous laser (Fig. S11). Incidentally, considering that the copper atoms diffuse from bulk to the Cu_2_O surface, the surface roughness increases, and the width of Cu_2_ONW thickens during oxidation.

In Fig. 2f, the SEM image of the seamless CuNW, Cu_2_ONW, and CuONW network demonstrates the morphology transmutation mentioned above. The inset image in Fig. 2f depicts a process diagram to fabricate a network parallelly arranged in three phases, where the left side red pseudocolor area is laser reduced CuNW, the middle yellow pseudocolor is Cu_2_ONW, and the right side black pseudocolor is laser-oxidized CuONW. The dotted line presents a nanowire including three phases Cu, Cu_2_O, and CuO simultaneously. This nanowire proves that CuONW is rougher and thicker than Cu_2_ONW. Figure 2g–i exhibits the magnified SEM image of CuNW, Cu_2_ONW, and CuONW, depicting surface roughness and thickness changes. The diameter increases to 120–150 nm during wet oxidation to become Cu_2_ONW. Then, through laser oxidation, the diameter of CuONW becomes 130–170 nm. CuNW becomes 80–110 nm again. Unlike the oxidation state, CuNW has a thinner nanowire thickness and low roughness. Furthermore, the nanowire network did not break using rSLIR, maintaining seamless interfacing remains even though the phase transition of synthesized CuNW occurred repeatedly.

### Reversible Oxidation State Control by rSLIR

**Fig. 3:**
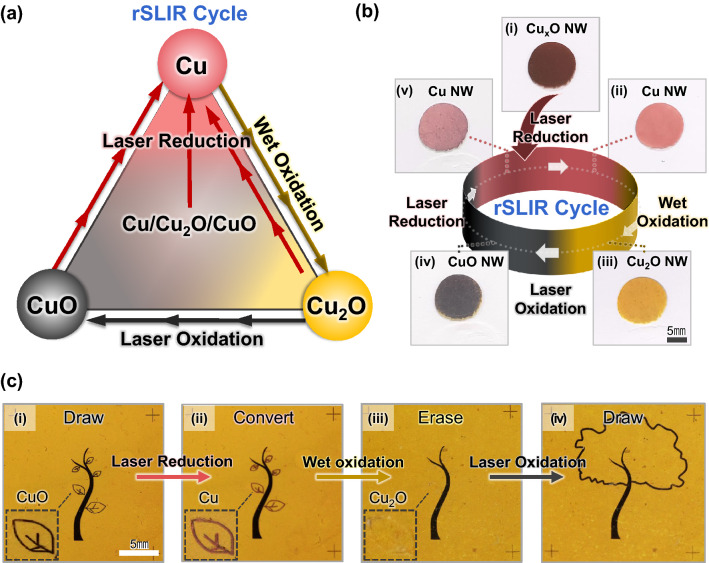
**a** A process diagram of rSLIR cycle; the arrows connect the reactive substance and the produced substance. **b** An optical image of rSLIR on the Cu_*X*_ONW network. **i** Prepared Cu*x*ONW network oxidized at 200 °C for 30 min. **ii** The first laser reduced CuNW. **iii** Wet-oxidized Cu_2_ONW. **iv** Laser-oxidized CuONW. **v** The second laser-reduced CuNW (laser oxidation, laser reduction conditions: 125 mW, 10 mm s^−1^, scale bar: 5 mm). **c** An optical image of rSLIR on the prepared pattern. **i** Yellow Cu_2_ONW with black CuONW tree pattern, and the inset optical image is a magnified black CuONW leaf. **ii** The leaves are converted to red CuNW through laser reduction. **iii** Removal of the pattern by wet oxidation. **iv** The new pattern is added to the existing pattern through laser oxidation (laser oxidation, laser reduction conditions: 125 mW, 10 mm s^−1^, scale bar: 5 mm)

rSLIR consists of wet oxidation, laser oxidation, and laser reduction and shows circularity when linking each process’s reactive and produced substances. Figure 3a shows that Cu can go back to its original oxidation state: Cu → Cu_2_O → CuO → Cu during rSLIR. Because of its unique properties, rSLIR ensures a high degree of freedom on the process sequence to fabricate a specific pattern. Any state in which Cu, Cu_2_O, and CuO single-phase and multi-phase coexist changes into the desired oxidation state. Figure 3b presents rSLIR to a Cu_*X*_ONW network. The dark-reddish Cu_*x*_ONW network is prepared by degradation at 200 °C for 30 min. According to the following process, the Cu_*X*_ONW network is sequentially transformed into a single-phase CuNW, Cu_2_ONW, and CuONW network: (1) Laser reduction changed the Cu_*X*_ONW network to a red CuNW network. (2) The CuNW network was converted to yellow Cu_2_ONW through wet oxidation and (3) became a black CuONW network by laser oxidation. (4) It returned to CuNW after going through laser reduction again.

Furthermore, customized re-writing is available by rSLIR. Figure 3c shows the seriate re-writing process, leaving the tree trunk intact, erasing only the leaves, and getting a new leafy pattern through the following steps: (1) Laser reduction converted the CuONW leaves pattern into CuNW. (2) The reduced CuNW became Cu_2_ONW by wet oxidation, which was identical to the yellow background. Since the wet oxidation was conducted under low temperature (100 °C), only CuNW suffered phase conversion to Cu_2_ONW. The remaining Cu_2_ONW and CuONW did not undergo a phase change during wet oxidation and maintained their color. (3) After that, laser oxidation fabricated a new black CuO pattern.

CuNW is susceptible to oxidation under ambient conditions due to its high surface-volume ratio, forming an irregular oxide layer and losing conductivity. For this reason, the reduction of nanomaterials has been a critical issue. Therefore, rSLIR employing circularity of the individual oxidation states will resolve the obstacle for broadening the uses for copper-based nanowire electronics. Furthermore, our method allows electrical property control through the cross-over of copper oxidation states, restores its conductivity, and functionalizes through oxidation state transition.

### Multispectral Photodetector Integration

The visible light photodetector is a foundational optoelectronic device. Moreover, its simple metal–metal oxide–metal (MSM) structure can replace the stacked-layer photodetector. Since the threshold of the light wavelength for generating a photocurrent is determined according to the bandgap of metal oxide, it is possible to easily fabricate visible-light photodetectors gaining a different wavelength threshold using multi-bandgap Cu_2_ONW and CuONW detecting channels with different bandgaps. In particular, illuminating with low energy red (650 nm, 1.9 eV) light excites electrons in CuO alone and has a relatively low bandgap. However, green (532 nm, 2.3 eV) and blue (450 nm, 2.8 eV) lights excite electrons in CuO and Cu_2_O. Here, we fabricated three types of photodetectors with varying responses according to wavelength and intensity through rSLIR.

**Fig. 4:**
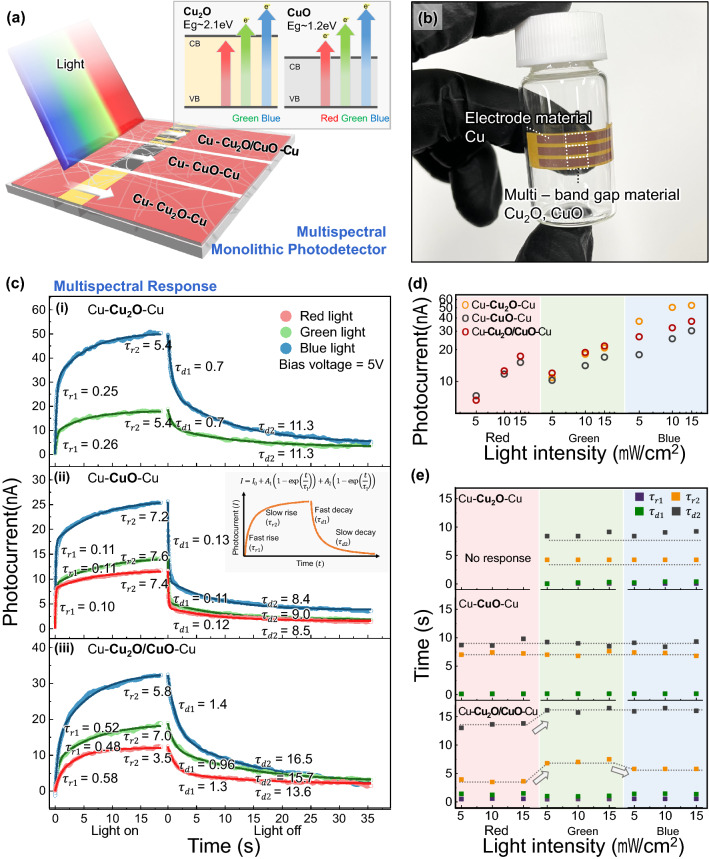
**a** The schematic depicts monolithic integration for multispectral photodetectors. The schematic shows the different wavelength thresholds caused by the bandgap difference between Cu_2_O and CuO. **b** PUA-nanowire composite flexible photodetector: red color CuNW as electrode region, and a detecting channel between the two electrodes. **c** Photocurrent and time constant according to 10 mW cm^−2^ light on or off, the inset schematic presents fast and slow relaxation constants of bi-exponential relaxation. **i** The Cu–Cu_2_O–Cu photodetector. **ii** Cu–CuO–Cu photodetector. **iii** Cu–Cu_2_O&CuO–Cu photodetector. **d** Log scale plot of the photocurrent magnitude at intensities of 5, 10, and 15 mW cm^−2^ for red (650 nm wavelength), green (532 nm wavelength), and blue light (450 nm wavelength). **e** Rise and decay constants at intensities of 5, 10, and 15 mW cm^−2^ for red (650 nm wavelength), green (532 nm wavelength), and blue light (450 nm wavelength)

Figure 4a illustrates the schematic of the three visible-light photodetectors, Cu–Cu_2_O–Cu, Cu–CuO–Cu, and Cu–Cu_2_O&CuO–Cu, fabricated by rSLIR. The Cu–Cu_2_O–Cu and Cu–CuO–Cu photodetectors consist of a single oxidation state detecting material, and their dimensions are 30 µm with a 2 mm height. Cu–Cu_2_O&CuO–Cu contains 40 parallel Cu_2_O and CuO channels with a width of 30 µm and a height of 50 µm. Moreover, we manufactured flexible photodetectors using simple polyurethane acrylate (PUA) embedded and detached from the glass substrate in Fig. 4b [43, 44]. These photodetectors show high oxidation-resistant property and bending durability (Fig. S12).

The behavior of the three types of photodetectors was confirmed by measuring the photocurrent response to red (650 nm, 1.9 eV), green (532 nm, 2.3 eV), and blue light (450 nm, 2.8 eV), and the moderate light intensity (5, 10, and 15 mW cm^−2^) under 5 V bias voltage, shown in Fig. S13. Usually, light illumination generates a photocurrent, and the photocurrent gradually rises and decays when the light is turned on or off.

The bi-exponential relaxation equation containing the fast (*A*_1_, *τ*_1_) and slow parts (*A*_2_, *τ*_2_) was used to analyze the gradual rise and decay curves for a quantitative approach.

2 $$ I = I_{0} + A_{1} \left( {1 - \exp \left( { - t/\tau_{1} } \right)} \right) + A_{2} \left( {1 - \exp \left( { - t/\tau_{2} } \right)} \right) $$

The relaxation time constant (*τ*_1,_ *τ*_2_) represents the curve shape, and the inset schematic depicts fast and slow relaxation. The quantitative analysis of the bi-exponential equation fitting shows a clear tendency for the illuminated light wavelength. Figure 4c shows the photocurrent curves divided into the rise and decay and the relaxation constants according to the red (650 nm, 1.9 eV), green (532 nm, 2.3 eV), and blue light (450 nm, 2.8 eV) illumination of each photodetector with 10 mW cm^−2^ intensity. The rise and decay constants of the Cu–Cu_2_O–Cu photodetector were estimated to be *τ*_r1_ = 0.25 s, *τ*_r2_ = 5.4 s, *τ*_d1_ = 0.7 s, and *τ*_d2_ = 11.3 s in green, *τ*_r1_ = 0.26 s, *τ*_r2_ = 5.4 s, *τ*_d1_ = 0.7 s, and *τ*_d2_ = 11.3 s in blue. The rise, decay constants for Cu–CuO–Cu are *τ*_r1_ = 0.10 s, *τ*_r2_ = 7.4 s, *τ*_d1_ = 0.12 s, and *τ*_d2_ = 8.5 s in red, *τ*_r1_ = 0.11 s, *τ*_r2_ = 7.6 s, *τ*_d1_ = 0.11 s, and *τ*_d2_ = 9.0 s in green, *τ*_r1_ = 0.11 s, *τ*_r2_ = 7.2 s, *τ*_d1_ = 0.13 s, and *τ*_d2_ = 8.4 s in blue. In these two types, the time constants barely changed regardless of the light wavelength. This consistency proceeds from the material’s inherent electron trap time, where the photon energy according to the wavelength has a minimal effect [45]. On the contrary, the time constants shift for the Cu–Cu_2_O&CuO–Cu photodetector since the Cu_2_O and CuO produce different signals.

In addition, to verify that this tendency was preserved with changes in luminous light intensity, we investigated the photocurrent magnitude and the time constants on all occasions. Figure 4d presents the photocurrent dependence on the light wavelength and intensity. As the light intensity increased, the photocurrent increased linearly on a log scale. The shorter the wavelength, the higher the photocurrent, even with the same intensity. As mentioned earlier, the bandgap of Cu_2_O is higher than red light photon energy, and only the Cu–Cu_2_O–Cu photodetector did not respond to red light in this range, yet it generated more photocurrent than other samples under green and blue light.

Figure 4e is the rise and decay constants according to wavelength and intensity. We demonstrated that the time constants of the Cu–Cu_2_O–Cu and Cu–CuO–Cu photodetectors remained nearly fixed while varying the wavelength and the power. This consistency means that a detecting channel with a single material requires additional measurement for the independent determination of wavelength and intensity. The time constants for the Cu–Cu_2_O&CuO–Cu photodetector, consisting of two detecting materials, shifted by wavelength, not intensity. Hence, this photodetector could be used to confirm the wavelength and intensity of the incident light by estimating the time constants and magnitude.

## Conclusion

We presented rSLIR as the first technology to obtain a monolithic CuNW, Cu_2_ONW, and CuONW network with a seamless interface. The conventional thermal oxidation method hinders selective patterning due to heat spreading, and it is incompatible with heat-susceptive substrates. Apart from other methods, our work achieved mask-less in situ oxidation state control under low temperature and ambient conditions. Moreover, this method induces the desired oxidation state regardless of the original state, restoring conductivity or imparting functionality. Experimental and theoretical analyses verified patterning performance and the Cu_2_O to CuO oxidation mechanism. A single nanowire with triple oxidation states indicates that rSLIR is a promising substitute for photolithography. Finally, rSLIR can be used to fabricate visible light photodetectors that generate varying photocurrents in accordance with wavelength and intensity. In this respect, our method works for controlling the electrical properties of transition metals. Thus we expect this technology to be exploited in various thin-film electronic devices.

## Supplementary Information

Below is the link to the electronic supplementary material.

Supplementary file 1 (PDF 1244 kb)
