# Supplementary material for: Multi-Bandgap Monolithic Metal Nanowire Percolation Network Sensor Integration by Reversible Selective Laser-Induced Redox
Source: Nanomicro Lett. 2022 Jan 25;14:49. doi: 10.1007/s40820-021-00786-1 (PMC8789997; doi:10.1007/s40820-021-00786-1)
Supplement: Supplementary file 1 — (PDF 1244 kb) [file 40820_2021_786_MOESM1_ESM.pdf]

Supplementary Information for

Multi-Bandgap Monolithic Metal Nanowire Percolation Network Sensor Integration by Reversible Selective Laser Induced Redox

Junhyuk Bang[1], Yeongju Jung[1], Hyungjun Kim[2], Dongkwan Kim[1], Maenghyo Cho[2], Seung Hwan Ko[1, 3, *]

[1] Applied Nano and Thermal Science Lab, Department of Mechanical Engineering, Seoul National University, 1 Gwanak-ro, Gwanak-gu, Seoul 151-742, Korea

[2] Department of Mechanical Engineering, Seoul National University, 1 Gwanak-ro, Gwanak-gu, Seoul, 08826, Republic of Korea

[3] Institute of Advanced Machines and Design / Institute of Engineering Research, Seoul National University, Seoul 08826, Korea

*Corresponding author. E-mail: maxko@snu.ac.kr (Seung Hwan Ko)

Supplementary Figures and Tables

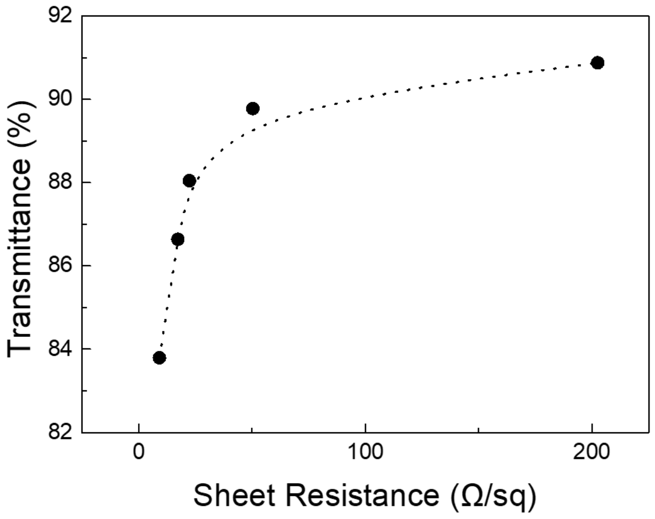

Fig. S1 Transmittance according to the sheet resistance of the CuNW

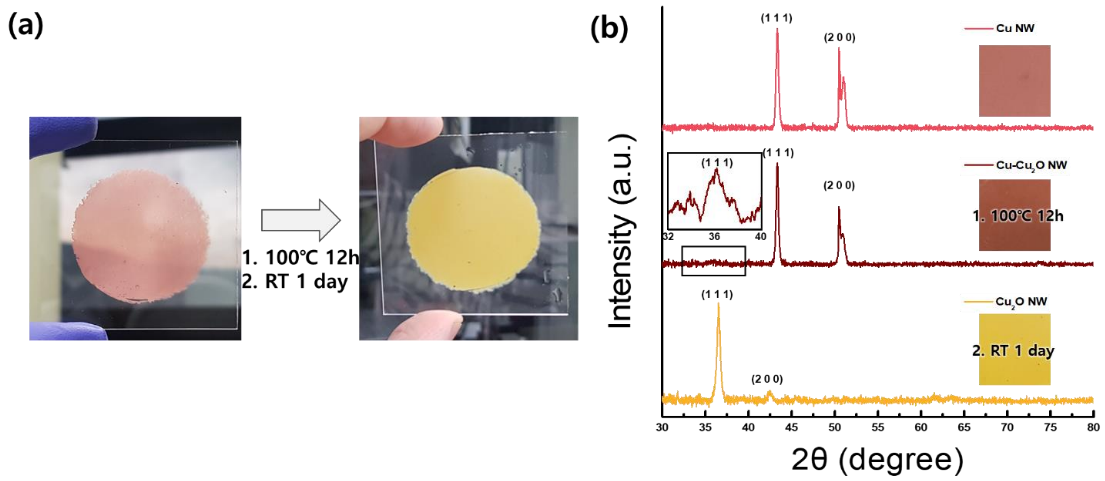

Fig. S2 Wet oxidation to CuNW to Cu_2ONW (**a**) the optical image of the red CuNW and the yellow Cu_2ONW, (**b**) XRD spectra during wet oxidation

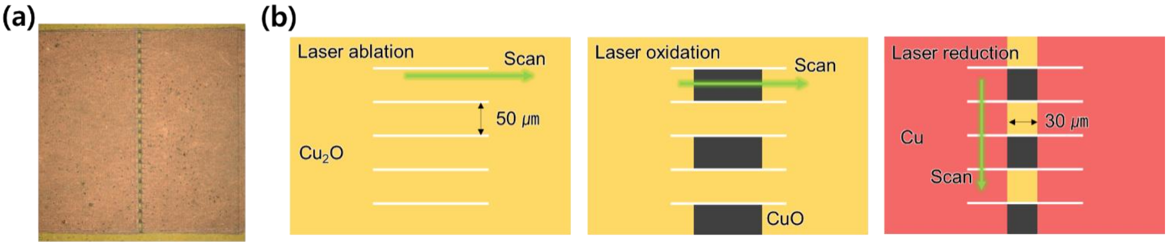

Fig. S3 (**a**)The optical image of Cu-Cu_2O&CuO-Cu photodetector. (**b**) Schematic depicting fabrication step: laser ablation → laser oxidation → laser reduction

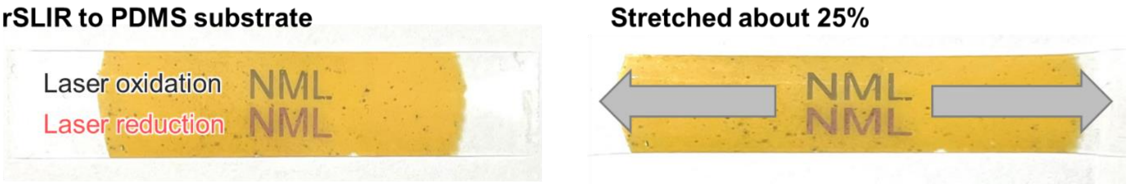

Fig. S4 rSLIR to stretchable PDMS substrate. We first fabricate Cu_2ONWs through wet oxidation. After that, CuONW and CuNW were patterned through laser oxidation and laser reduction under the conditions of 70 mW of laser power and 10 mm/s of scanning speed

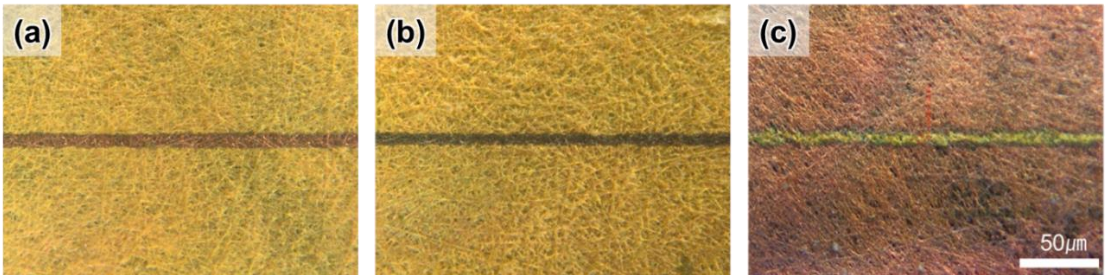

Fig. S5 Resolution of Cu-based material pattern. (**a**) CuNW, (**b**) CuONW, and (**c**) Cu_2ONW

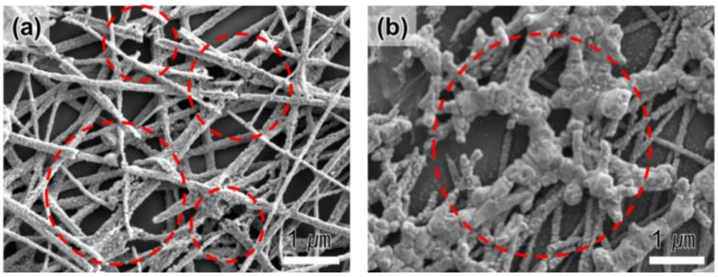

Fig. S6 High power density laser illumination. (**a**) laser reduction, (**b**) laser oxidation

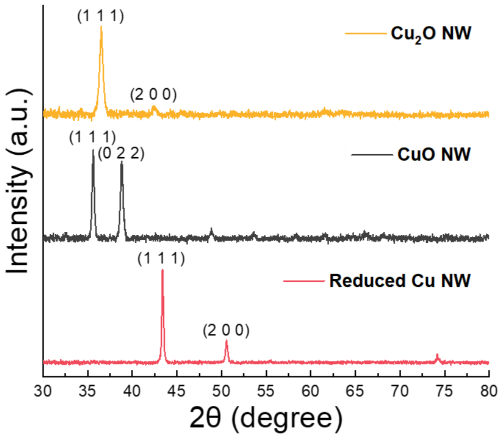

Fig. S7 XRD spectra of Cu_2ONW, CuONW, and reduced CuNW prepared on glass substrates

Table S1 FWHM for the (111) peak for XRD data for Cu_2ONW and CuONW

NW	XRD FWHM for (111)
Cu_2O	0.408
CuO	0.266

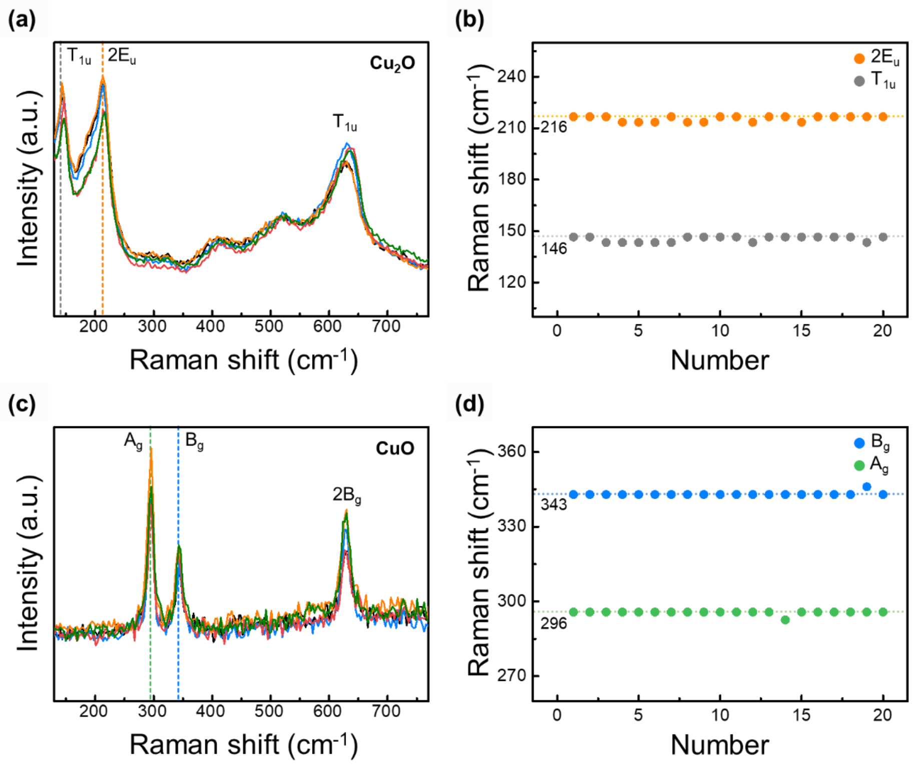

Fig. S8 Repeatability of Cu_2ONW and CuO NW. (**a**) The overlapped Raman spectrum measured at 5 points without intensity calibration. All results show similar inclination, peaks, and intensities. (**b**) The main peaks (T_{1u} : 146 cm^{-1}, $2E_u$: 216 cm^{-1}) of the Raman spectrum at random 20 points of Cu_2ONW. (**c**, **d**) The same analysis results for CuONW. (A_g : 296 cm^{-1} , B_g : 343 cm^{-1})

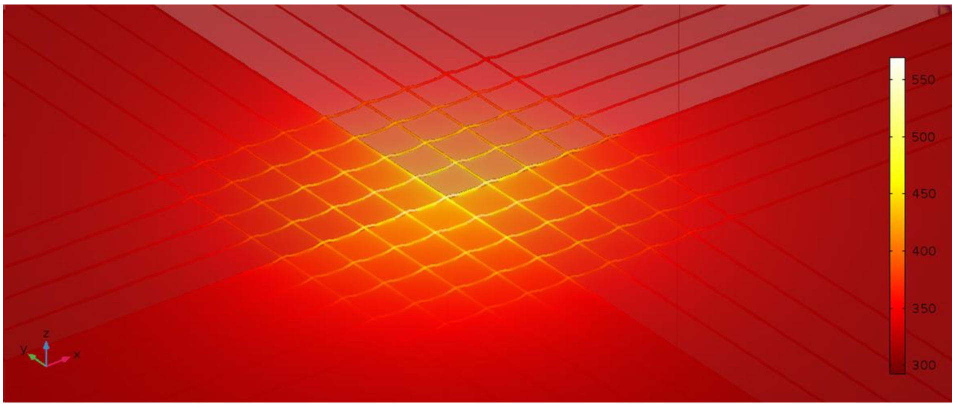

Fig. S9 Comsol image of Finite element method simulation at laser illumination to nanowires network (beam spot size of 20 µm and a power of 40 mW)

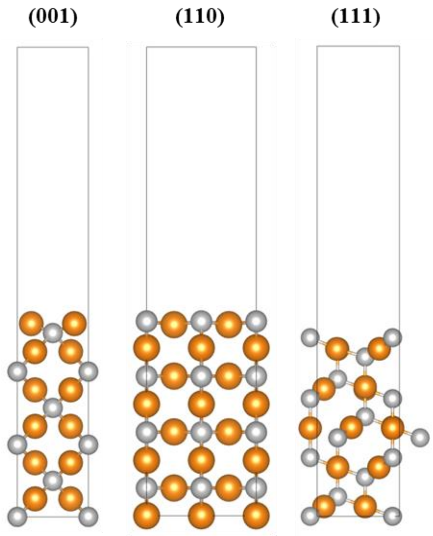

Fig. S9 The slab models of the (001), (110), and (111) planes of Cu_2O for DFT calculation of surface energy

Table S2 The surface energy for the (001), (110), and (111) planes of Cu_2O

Surface	Surface energy (J/m^2)
(001)	1.318
(110)	1.159
(111)	0.772

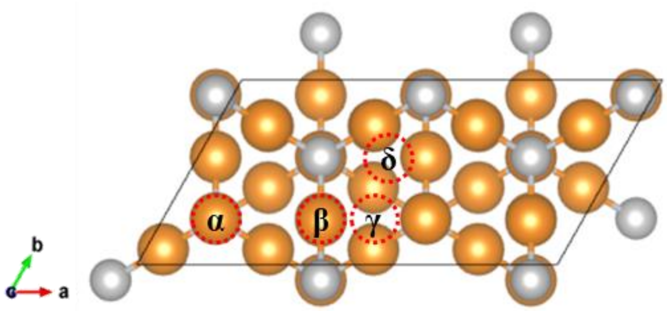

Fig. S10 The possible O_2 molecule adsorption sites on Cu_2O(111) surface

Table S3 The adsorption energy for the possible O_2 molecule adsorption sites on Cu_2O(111) surface

Adsorption site	Adsorption energy (eV)
α	-1.068
β	-0.438
γ	-1.156
δ	-1.067

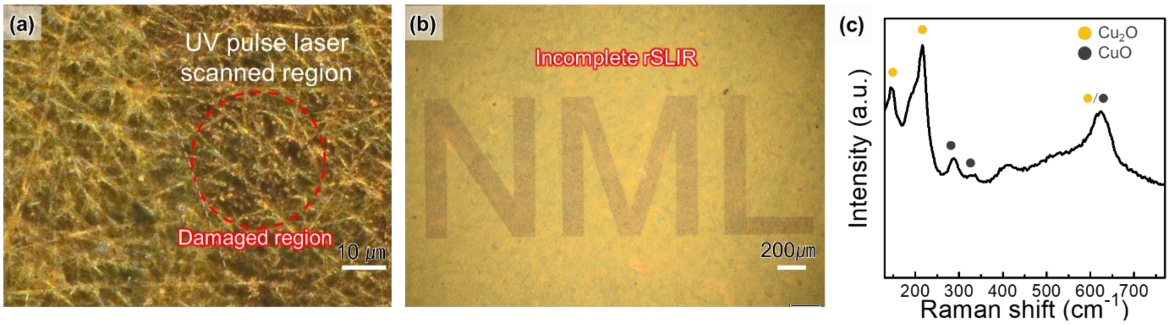

Fig. S11 Pulsed laser rSLIR. (**a**) An optical image of the nanowire network after ten cycles of hatch scanning at 1.68 mW. Unlike continuous laser oxidation, it does not change to black color, and even nanowires are ablated and damaged. (**b**) Pulsed laser oxidation with slightly lower power. complete oxidation does not occur even with 20 laser scanning cycles. (**c**) Raman spectrum of incomplete laser oxidation

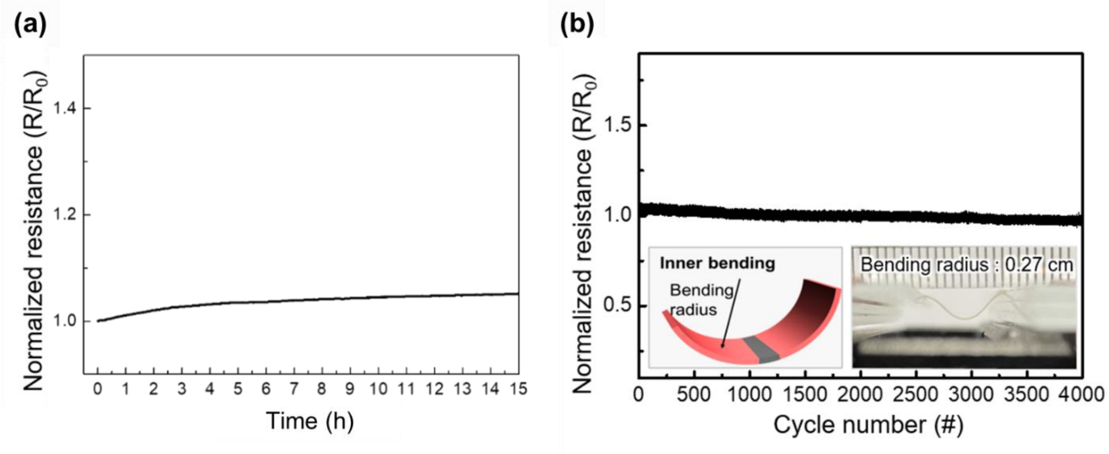

Fig. S12 (**a**) Resistant change of CuNW network during 85/85test. (**b**) Cyclic bending test at bending radius of 0.27cm

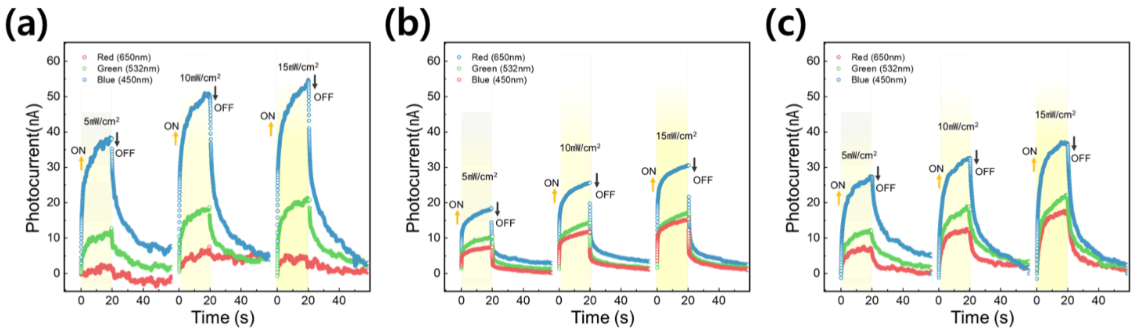

Fig. S13 The behaviors of three types of photodetectors varying luminous light wavelength and intensity (**a**) Cu-Cu_2O-Cu photodetector, (**b**) Cu-CuO-Cu photodetector, and (**c**) Cu-Cu_2O&CuO-Cu photodetector
